# Transplant experiments uncover Baltic Sea basin-specific responses in bacterioplankton community composition and metabolic activities

**DOI:** 10.3389/fmicb.2015.00223

**Published:** 2015-04-01

**Authors:** Markus V. Lindh, Daniela Figueroa, Johanna Sjöstedt, Federico Baltar, Daniel Lundin, Agneta Andersson, Catherine Legrand, Jarone Pinhassi

**Affiliations:** ^1^Centre for Ecology and Evolution in Microbial Model Systems, Linnaeus University, KalmarSweden; ^2^Department of Ecology and Environmental Science, Umeå University, UmeåSweden; ^3^Umeå Marine Sciences Centre, Umeå University, UmeåSweden; ^4^Department of Marine Science, University of Otago, DunedinNew Zealand

**Keywords:** bacterial community functioning, salinity, DOM, terrigenous carbon, climate change, marine bacteria, bacterial diversity

## Abstract

Anthropogenically induced changes in precipitation are projected to generate increased river runoff to semi-enclosed seas, increasing loads of terrestrial dissolved organic matter and decreasing salinity. To determine how bacterial community structure and functioning adjust to such changes, we designed microcosm transplant experiments with Baltic Proper (salinity 7.2) and Bothnian Sea (salinity 3.6) water. Baltic Proper bacteria generally reached higher abundances than Bothnian Sea bacteria in both Baltic Proper and Bothnian Sea water, indicating higher adaptability. Moreover, Baltic Proper bacteria growing in Bothnian Sea water consistently showed highest bacterial production and beta-glucosidase activity. These metabolic responses were accompanied by basin-specific changes in bacterial community structure. For example, Baltic Proper *Pseudomonas* and *Limnobacter* populations increased markedly in relative abundance in Bothnian Sea water, indicating a replacement effect. In contrast, *Roseobacter* and *Rheinheimera* populations were stable or increased in abundance when challenged by either of the waters, indicating an adjustment effect. Transplants to Bothnian Sea water triggered the initial emergence of particular *Burkholderiaceae* populations, and transplants to Baltic Proper water triggered *Alteromonadaceae* populations. Notably, in the subsequent re-transplant experiment, a priming effect resulted in further increases to dominance of these populations. Correlated changes in community composition and metabolic activity were observed only in the transplant experiment and only at relatively high phylogenetic resolution. This suggested an importance of successional progression for interpreting relationships between bacterial community composition and functioning. We infer that priming effects on bacterial community structure by natural episodic events or climate change induced forcing could translate into long-term changes in bacterial ecosystem process rates.

## Introduction

A fundamental question in ecology focuses on whether shifts in diversity and community composition due to changes in environmental conditions also result in changes in bacterial community functioning (Loreau, 2000; Gamfeldt and Hillebrand, 2008). Overall, little is known about how bacterial community composition affects bacterial community functioning and how sensitive or resistant bacterial communities and individual taxa are to environmental disturbances (Allison and Martiny, 2008; Comte and Del Giorgio, 2011). It is, therefore, desirable to examine the adaptability (i.e., sensitivity, resistance, and responsiveness) and metabolic plasticity (i.e., the potential to achieve similar ecosystem process rates) of bacterioplankton populations responding to environmental disturbances. Most bacterial populations are sensitive to environmental disturbances, and changes in bacterial community composition can influence the rates of ecosystem processes, suggesting that populations are functionally dissimilar (Bell et al., 2005; Langenheder et al., 2005; Judd et al., 2006; Allison and Martiny, 2008; Comte and Del Giorgio, 2011; Comte et al., 2013). However, little is known about the changes in population dynamics and ecosystem ecology in response to climate change consequences, such as increased temperature, lower pH, or increased river runoff (Degerman et al., 2013; Lindh et al., 2013; Von Scheibner et al., 2014). Potentially, knowledge of the responses of bacterioplankton populations to anthropogenically induced environmental change could extend the understanding of the links between population dynamics and ecosystem ecology and might help to predict and monitor future change in marine environments.

Projections from climate change models highlight increased annual levels of precipitation in Northern Europe, decreasing salinity and increasing loadings of terrigenous (allochthonous) dissolved organic matter (DOM) to coastal waters through river outflows (Meier, 2006). Changes in salinity and increased terrigenous carbon inputs have been shown to influence growth and activity of bacterioplankton (del Giorgio and Bouvier, 2002; Langenheder et al., 2003; Kritzberg et al., 2004; Rochelle-Newall et al., 2004; Laghdass et al., 2010; Fasching et al., 2014). Salinity is an important factor shaping bacterial community composition in that it influences the spatial distribution of bacterial populations (Lozupone and Knight, 2007; Herlemann et al., 2011; Dupont et al., 2014). On the other hand, bacterial community composition is also much dependent on the quantity and quality of DOM (Lindström, 2000; Eiler et al., 2003; Kisand and Wikner, 2003; Kirchman et al., 2004; Rochelle-Newall et al., 2004; Kritzberg et al., 2006; Kisand et al., 2008; Teira et al., 2009; Gómez-Consarnau et al., 2012; Grubisic et al., 2012; Rocker et al., 2012). Yet, empirical data for how bacterial community functioning and the cycling of carbon will be affected in coastal or semi-enclosed waters like the Baltic Sea under conditions simulating potential future climate change influences are scarce. Detailed knowledge on the combined effects of climate change driven changes in salinity and DOM for bacterial community composition and metabolic activity would be desirable.

As a semi-enclosed sea, the Baltic Sea is characterized by seasonally changing inputs in the quality and quantity of allochthonous DOM (Zweifel et al., 1993). In addition, the prominent salinity gradient ranges from truly marine in the southern to freshwater salinities in the northern basins of the Baltic Sea, where large river discharges cause lower salinity. The cause for differences in the distribution of microbial populations due to salinity is likely related to the long residence time in the Baltic Sea (>5 years), allowing niche differentiation and adaptions to optimum salinity levels (Riemann et al., 2008; Herlemann et al., 2011; Dupont et al., 2014). The combined environmental disturbances projected from climate change models imply substantial effects on the structure and function of both macro- and microorganism communities, including bacterioplankton, in the Baltic Sea (Wikner and Andersson, 2012). One of the major consequences of such anthropogenically induced disturbances for marine microbes is expected to be a change in biogeochemical cycling of carbon that may allocate more energy for heterotrophic bacterial production in the Baltic Sea (Sandberg et al., 2004; Wikner and Andersson, 2012).

**FIGURE 1:**
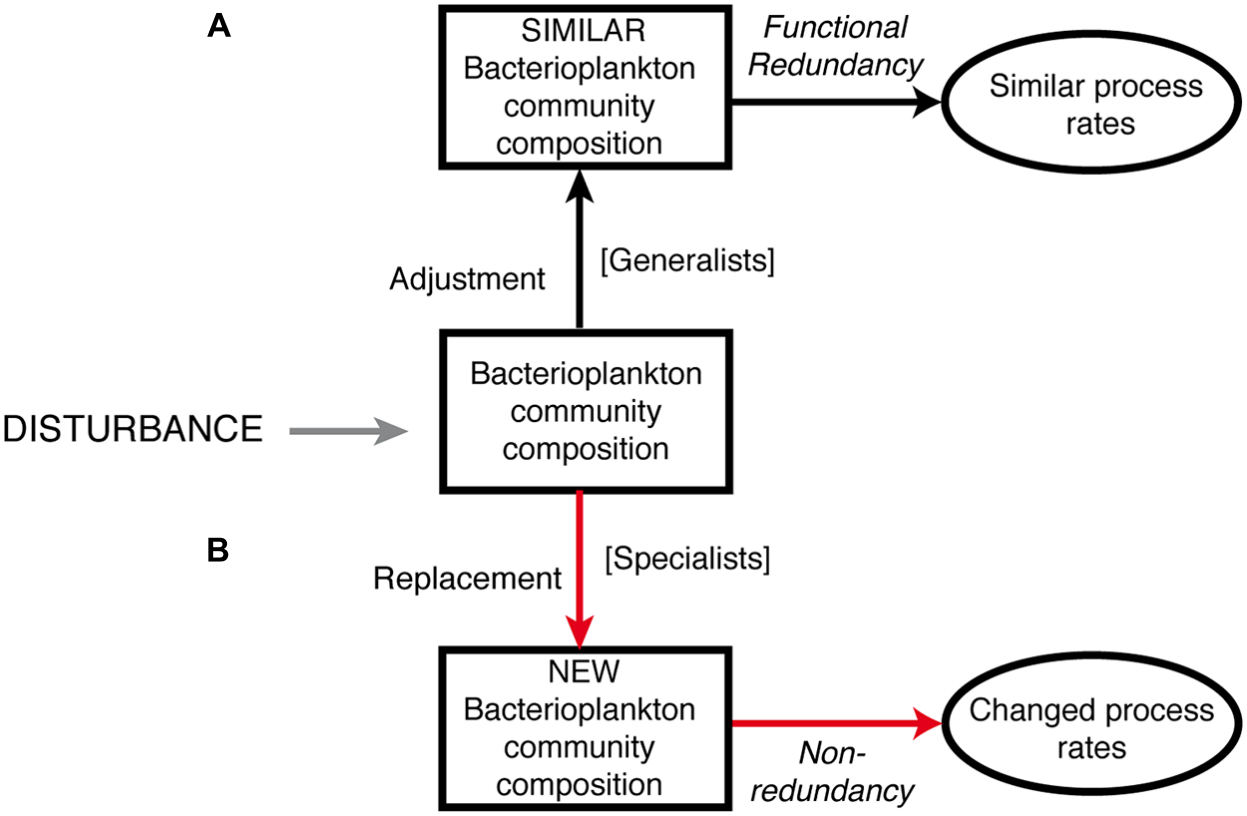
**Conceptual model of the potential outcome of this study.** We hypothesized that bacterioplankton responses in community composition and metabolic activity would follow pathway **B**; red arrows, i.e., replacement of OTUs, leading to changes in community composition and functioning. Our null-hypothesis is therefore pathway **A**; black arrows; i.e., adjustment of OTUs, leading to unchanged community composition and bacterial community functioning.

Transplant experiments have provided insights into key factors that regulate bacterial community structure, diversity, and functioning in different aquatic environments (Gasol et al., 2002; Kirchman et al., 2004; Rochelle-Newall et al., 2004; Langenheder et al., 2005; Bonilla-Findji et al., 2009; Sjöstedt et al., 2012; Comte et al., 2013). The aim of the present study was to investigate how the quality of water originating from geographically distinct basins of the Baltic Sea influences bacterial community composition and metabolism. This was done under the premise that projections of future climate change influence on the Baltic Sea indicate that increased precipitation will lead to environmental conditions in the Baltic Proper similar to those currently found in the northern basins of the Baltic Sea (Bothnian Sea or Bothnian Bay). We designed a transplant and re-transplant microcosm experiment and monitored the effects on bacterial community composition (by using 16S rRNA gene Illumina Miseq tag sequencing) and functioning (i.e., bacterial abundance, production, and enzyme activities). A conceptual model of potential outcomes of this study is presented in **Figure 1**. We hypothesized that: (i) bacterial community composition would change after both transplantation and re-transplantation disturbances relative to controls following the replacement scenario (pathway B in **Figure 1**) *sensu* Allison and Martiny (2008) and Comte and Del Giorgio (2011) and (ii) bacterial community functioning would be affected due to limited functional redundancy.

## Materials and Methods

### Field Sampling

**FIGURE 2:**
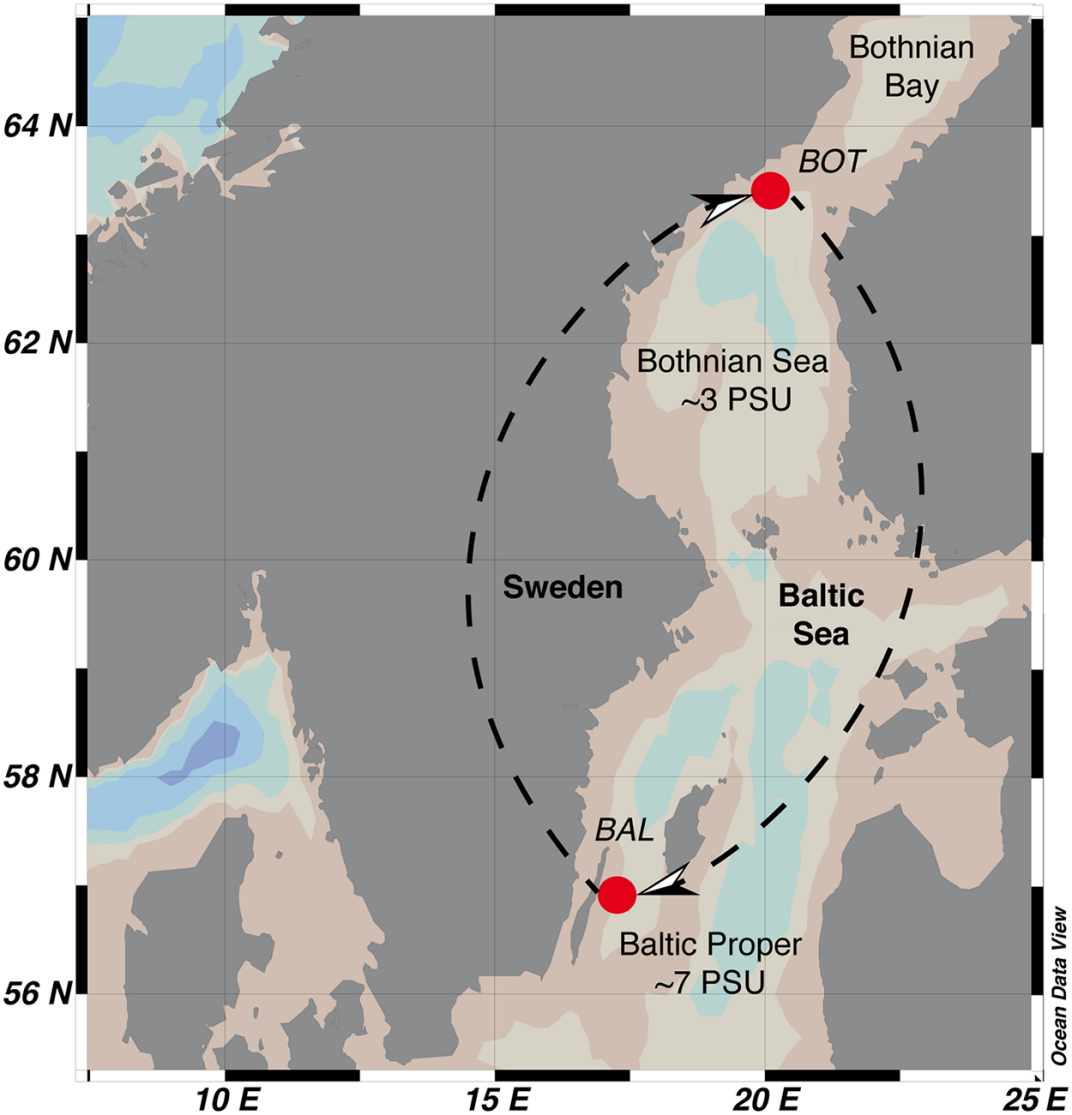
**Geographic location of the Baltic Sea Proper (BAL) and Bothnian Sea (BOT) stations in the Baltic Sea.** Arrows indicate direction of transplant and re-transplant of unfiltered seawater (inoculum) to seawater media.

Culture media for the experiments was prepared from seawater collected from the Baltic Sea Proper (BAL; Linnaeus Microbial Observatory station, LMO; N 56°55.851, E 17°03.640) and the Bothnian Sea (BOT; NB1; N 63°31.0000, E 19°48.1166) on the 1 and 2 July 2013, respectively (**Figure 2**). Seawater was taken using a Ruttner sampler at a depth of 2 m. BAL and BOT water were transported in the dark to the laboratory in acid-washed Milli-Q rinsed polycarbonate bottles and at *in situ* temperatures within 1 or 12 h, respectively.

Seawater to be used as inoculum with natural bacterioplankton assemblages was collected simultaneously at the BAL and BOT sites on 15 July 2013, and was transported to the Linnaeus University within 12 h. This seawater for inocula remained unaltered (i.e., no manipulations such as filtrations were carried out). On both field samplings, measurements of temperature, salinity, and nutrient concentrations were taken. For the second field sampling, when water for bacterial inocula was obtained, nutrient limitation bioassays were carried out, and samples for determining *in situ* bacterial community composition were collected.

### Microcosm Setup

Water from each of the two stations was prepared for seawater culture media by sterile filtration (0.2 μm pore size; Sterivex cartridge; Millipore, USA), whereupon the filtrate was distributed into acid-washed Milli-Q rinsed 2 l polycarbonate bottles followed by autoclaving and subsequent storage in the dark at 16°C. Prior to inoculation, culture media had <10^4^ cells ml^-1^, as determined by flow cytometry. The experiment was made up of two parts: a transplant and a re-transplant part, running 5 and 4 days, respectively. In the transplant, unfiltered seawater was used to inoculate the sterile filtered and autoclaved seawater media in triplicates for each treatment at a ratio of 1:20. For the re-transplant, inoculum from transplant microcosms on day 5 were added to sterile filtered autoclaved seawater media in triplicates at a ratio of 1:20. This ratio was used based on our previous experience in obtaining clear bacterial growth responses in Baltic Sea microcosms (Gómez-Consarnau et al., 2012). Nomenclature of microcosms is as follows; station_b_ → station_sw_ where subscript “b” indicates bacteria and subscript “sw” indicates seawater medium. Thus, transplant microcosms consisted of native Baltic Proper bacteria growing in either Baltic Proper water (BAL_b_ → BAL_sw_) or Bothnian Sea water (BAL_b_ → BOT_sw_), and native Bothnian Sea bacteria incubated in either Bothnian Sea water (BOT_b_ → BOT_sw_) or Baltic Proper water (BOT_b_ → BAL_sw_). Re-transplant microcosms consist of Baltic Proper bacteria re-transferred to Baltic Proper water (BAL_b_ → BOT_sw_ → BAL_sw_) or with continued growth in Bothnian Sea water (BAL_b_ → BOT_sw_ → BOT_sw_), and Bothnian Sea bacteria re-transferred to Bothnian Sea water (BOT_b_ → BAL_sw_ → BOT_sw_) or continued incubation in Baltic Proper water (BOT_b_→BAL_sw_→BAL_sw_). All microcosms were incubated at 16°C in darkness. The microcosms were gently inverted manually twice a day and before sampling of biotic and abiotic parameters. The experimental setup is summarized in **Table 1** and detailed in Figure S1. In the transplant experiment, salinity was measured daily and total organic carbon (TOC) concentrations were measured on day 0, 2, and 5 (triplicates). Bacterial abundance was monitored daily by flow cytometry (duplicates) and heterotrophic production was determined on day 0, 2, and 4 (quadruplicates). Extracellular enzyme activities were measured on day 0, 2, and 4 (quadruplicates). In the re-transplant experiment, salinity was measured daily and TOC concentrations were measured on day 0, 2, and 4 (triplicates). Bacterial abundance (duplicates) and production (quadruplicates) were measured daily and extracellular enzyme activities were measured on day 0, 2, and 3 (quadruplicates).

**Table 1:** Simplified experimental setup of the microcosm experiment.

	*Seawater media*			
*Bacterial source*	Transplant	Transplant control	Re-transplant	Re-transplant control
BAL_b_	BOT_sw_	BAL_sw_	BAL_sw_	BOT_sw_
BOT_b_	BAL_sw_	BOT_sw_	BOT_sw_	BAL_sw_

Unfiltered water sampled in the Baltic Sea proper (BAL) and in the Bothnian Sea (BOT) was inoculated in triplicates at 1:20 ratio into sterile filtered autoclaved seawater media from these two stations. For re-transplant, samples from transplant microcosms were inoculated in triplicates at 1:20 ratio.

### Nutrient Concentrations

*In situ* samples from the BAL and BOT stations for dissolved inorganic nutrient concentrations (NH_4_^+^, NO_3_^-^, PO_4_^3-^) were collected on the 15 July, when the water for the inocula was sampled, and were analyzed following the method of Valderrama (1995). For measuring TOC concentration, samples of 50 ml were filtered (0.2 μm Supor Membrane Syringe Filter, non-pyrogenic; Acrodisc®; Pall Life Sciences, USA), acidified with 0.67 ml of 1.2 M HCl and kept in acid rinsed 50 ml Falcon tubes at 4°C in the dark until processing. The samples were purged and measured using a Shimadzu TOC-5000 Analyzer (Shimadzu, Japan).

### Bacterial Abundance, Bacterial Heterotrophic Production, and Extracellular Enzyme Activity

Bacterial abundance samples of 900 μl were preserved with formaldehyde (2% final concentration) and stored at -80°C until processing. Bacterial abundance was measured by staining samples with SYTO 13 (5 μM final concentration: Molecular Probes, USA) and enumerated using a Cube 8 flow cytometer (Partec, Germany) according to the protocol described in del Giorgio et al. (1996). For bacterial heterotrophic production, 1.2 ml samples were collected with two killed controls and production was measured via the ^3^H-Leucine method according to Smith and Azam (1992). Extracellular enzymatic activities of beta-glucosidase, leucine aminopeptidase, and alkaline phosphatase were determined in technical quadruplicates according to the fluorometric enzyme assays described in Baltar et al. (2010).

### Nutrient Limitation Bioassays

Bacterial nutrient limitation was measured for *in situ* seawater by distributing 250 ml of seawater to acid-washed Milli-Q rinsed polycarbonate bottles adding 24 μM glucose (C_6_H_12_O_6_), 4.2 μM ammonium (NH_4_Cl), and 0.6 μM phosphate (NaH_2_PO_4_; final concentrations) in duplicate treatments incubating in the dark for 48 h at 16°C. Differential responses to nutrient addition were determined by measuring bacterial heterotrophic production.

### DNA and Illumina Miseq PCR

Collection of biomass for DNA extraction was done on day 5 for the transplant and day 4 for the re-transplant when 750 ml of water was filtered onto 0.2 μm 47 mm Supor filters (PALL Life Sciences) for all treatments except for the *in situ* samples for which 4 l were Sterivex filtered (Millipore). Phenol-chloroform extraction of DNA was performed according to Riemann et al. (2000). Bacterial 16S rRNA was first amplified with HPLC purified bacterial primers 341F and 805R (Herlemann et al., 2011) following the PCR protocol of Hugerth et al. (2014) with some modifications; amplification was carried out in duplicates for each biological replicate and we used an annealing temperature of 58°C in the first PCR and 12 cycles in the second PCR. The resulting purified amplicons were sequenced on the Illumina Miseq (Illumina, USA) platform using the 300 bp paired-end setting at the Science for Life Laboratory, Sweden (www.scilifelab.se). Due to problems with either sampling or sequencing some treatments are only represented by duplicates or a single sample (Table S1).

### Sequence Processing and Analysis

Raw sequence data generated from Illumina Miseq were processed using the UPARSE pipeline (Edgar, 2013). Taxonomy was determined against the SINA/SILVA database (SILVA 115; Quast et al., 2013). After quality control, our data consisted of a total of 1.3 million reads, with an average of 40 086 ± 18 037 reads per sample. These sequences resulted in a final OTU table consisting of 3920 OTUs (excluding singletons) delineated at 97% 16S rRNA gene identity. For the OTU based analyses, chloroplast, mitochondrial, and eukaryotic sequences have been excluded from all analyses. The maximum likelihood tree was made using MEGA 5.2.1 and the Tamura-Nei model (Tamura et al., 2011) to examine the phylogenetic relationship between bacterioplankton responding in different microcosms and for Unifrac analysis. DNA sequences have been deposited in the National Center for Biotechnology Information (NCBI) Sequence Read Archive under accession number SRP048666.

### Statistical Analyses

For analysis of variance (ANOVA) statistics we tested the sample distribution for normality using Shapiro tests, and if the data was not normally distributed we log-transformed the data. ANOVA results were complemented with Tukey’s *post hoc* test. To investigate patterns of bacterial community composition, non-metric multidimensional scaling (nMDS, Bray–Curtis distance) ordination and UPGMA (unweighted pair group arithmetic mean, UniFrac distance) dendrogram were used. Unifrac analysis was based on the average relative abundance of replicate microcosms. Differences in community composition between microcosms were tested using permutational analysis of variance (PERMANOVA) on Bray–Curtis distances. In our detailed OTU analyses (**Figure 7**; **Table 2**) we first selected the 200 most abundant OTUs that is OTUs with the highest total relative abundance across the experiments. These OTUs together represented 82% of total sequence reads. We further examined in detail the response in our transplant experiments of bacterial OTUs that typically represent abundant populations in the Baltic Sea (see, e.g., Herlemann et al., 2011; Lindh et al., 2015). Pronounced responses of particular OTUs were determined by comparing changes in relative sequence abundance between treatments and experiments. Correlations between community composition and enzymatic activity for different taxa were tested using PERMANOVA with Bray–Curtis distances. For testing the correlation between changes in community functioning and shifts in bacterioplankton community composition we performed MANTEL tests. We, therefore, combined the differential response of bacterial production and enzyme activities between microcosm treatments and constructed a distance matrix using the Canberra distance estimation. This community functioning matrix was compared with Bray–Curtis dissimilarity matrices of community composition at different cluster levels (99, 97, 95, 93, and 91% 16S rRNA gene identity). All statistical tests were performed in R 3.0.2 (R Core Team, 2014), using the packages Vegan (Oksanen et al., 2010) and Picante (Kembel et al., 2010). Graphical outputs were made using the package ggplot2 (Wickham, 2009).

**Table 2:** Relative abundances of important OTUs (delineated at 97% 16S rRNA gene identity) found during the experiments.

	OTU	Taxon (highest identified taxonomic rank)	Phyla/Class	BAL_b_ →BAL_sw_	BOT_b_ →BOT_sw_	BOT_b_ →BAL_sw_	BAL_b_ →BOT_sw_	BAL_b_ →BOT_sw_ →BAL_sw_	BAL_b_ →BOT_sw_ →BOT_sw_ §	BOT_b_ →BAL_sw_ →BOT_sw_	BOT_b_ →BAL_sw_ →BAL_sw_
*In situ*	TR_000038	CL500-29	Actino.	–	0.3 (0.4)	0.2 (0.3)	-	0.1 (0.1)	-	–	–
	TR_000029	hgcI clade	Actino.	0.1 (0.3)	0.3 (0.4)	0.2 (0.3)	0.4 (0.4)	–	–	–	–
	TR_000037	SAR11 clade	Alpha.	<0.1 (0.1)	0.3 (0.3)	0.2 (0.3)	0.1 (0.2)	–	–	–	–
	TR_000014	*Roseobacter* clade	Alpha.	5.2 (10.5)	0.2 (0.3)	0.3 (0.4)	2.7 (3.0)	0.3 (0.6)	–	0.2 (0.3)	0.4 (0.4)
	TR_000036	NS3a	Bact.	<0.1 (0.1)	–	–	–	–	–	–	–
	TR_000133	*Polaribacter*	Bact.	0.2 (0.3)	–	0.1 (0.1)	0.1 (0.1)	<0.1 (0.1)	–	–	<0.1 (0.1)
	TR_000076	BAL58	Beta.	<0.1 (0.1)	–	<0.1 (0.1)	0.1 (0.1)	–	–	–	–
	TR_000025	*Synechococcus*	Cyano	<0.1 (0.1)	0.1 (0.1)	<0.1 (0.1)	0.1 (0.1)	–	–	–	–
	TR_000055	SAR86 clade	Gamma	–	–	–	–	–	–	–	–
	TR_000020	*LD29*	Verr.	0.1 (0.1)	0.1 (0.1)	–	0.2 (0.3)	–	–	–	–
BAL_b_	TR_000018	*Seohaeicola^∗^*	Alpha.	3.6 (7.3)	<0.1 (0.1)	0.4 (0.6)	1.1 (1.2)	5.4 (8.4)	0.5	0.7 (0.9)	2.7 (2.9)
	TR_000033	*Brevundimonas^∗^*	Alpha.	–	–	–	–	2.4 (4.8)	4.5	–	–
	TR_000200	*Litoreibacter*	Alpha.	5.7 (11.5)	0.2 (0.2)	0.4 (0.5)	1.8 (2.2)	0.3 (0.5)	0.0	0.2 (0.3)	0.7 (0.8)
	TR_000014	see *in situ* above									
	TR_005668	*Limnobacter^∗^*	Beta.	0.1 (0.2)	–	–	0.8 (1.2)	7.0 (10.0)	10.1	0.8 (1.6)	–
	TR_000005	*Limnobacter^∗^*	Beta.	0.1 (0.1)	–	–	0.7 (1.1)	6.5 (9.6)	8.1	0.8 (1.6)	–
	TR_009032	*Limnobacter^∗^*	Beta.	<0.1 (0.1)	–	–	0.7 (1.1)	5.0 (6.8)	7.8	0.7 (1.5)	–
	TR_000010	*Pseudomonas*	Gamma.	–	–	<0.1 (0.1)	1.2 (2.2)	15.2 (22.1)	24.5	1.9 (3.7)	–
	TR_000006	*Rheinheimera*	Gamma.	26.8 (53.6)	7.1 (8.4)	4.9 (7.9)	36.2 (37.5)	15.0 (28.1)	2.5	9.2 (14.3)	2.0 (2.4)
	TR_002843	*Rheinheimera*	Gamma.	9.4 (18.8)	1.2 (1.7)	1.9 (2.5)	2.7 (3.3)	0.3 (0.4)	0.1	0.8 (1.1)	0.9 (1.0)
BOT_b_	TR_000003	*Loktanella*	Alpha.	1.5 (1.8)	7.2 (14.4)	5.6 (6.3)	2.1 (2.4)	0.4 (0.9)	–	3.4 (5.5)	10.6 (11.4)
	TR_000019	*Rhodobacteraceae*	Alpha.	2.4 (2.4)	0.1 (0.3)	2.3 (3.4)	0.3 (0.4)	<0.1 (0.1)	–	0.6 (0.7)	1.3 (1.5)
	TR_000012	*Alishewanella*	Gamma.	–	0.1 (0.1)	0.4 (1.2)	–	–	–	2.1 (3.9)	2.7 (2.9)
	TR_008541	*Pseudomonas^∗^*	Gamma.	–	2.3 (2.7)	3.1 (4.1)	–	–	–	2.8 (3.0)	2.6 (2.7)
	TR_007801	*Pseudomonas^∗^*	Gamma.	–	2.0 (2.4)	2.7 (3.6)	–	–	–	2.5 (2.6)	1.9 (1.9)
	TR_000001	*Pseudomonas^∗^*	Gamma.	–	1.5 (1.8)	1.8 (2.4)	–	–	–	1.7 (1.7)	1.4 (1.4)
	TR_002653	*Rheinheimera^∗^*	Gamma.	<0.1 (0.1)	0.3 (0.6)	4.3 (4.6)	–	<0.1 (0.1)	0.1	4.7 (7.6)	7.0 (8.3)
	TR_000007	*Rheinheimera*	Gamma.	–	0.3 (0.5)	3.3 (3.4)	–	<0.1 (0.1)	0.1	3.7 (6.3)	5.5 (6.2)
	TR_001027	*Rheinheimera^∗^*	Gamma.	–	0.2 (0.4)	2.1 (2.6)	–	–	–	2.6 (4.4.)	3.3 (4.2)
	TR_000820	*Rheinheimera^∗^*	Gamma.	–	0.2 (0.3)	1.5 (1.8)	–	–	–	1.4 (2.5)	1.5 (1.7)

Responses of usually numerically abundant Baltic Sea OTUs that were also dominant in the *in situ* samples in this study, followed by the 10 most abundant Baltic Proper OTUs with the largest difference in relative abundance compared to Bothnian Sea populations and top 10 Bothnian Sea OTUs with the largest difference in relative abundance compared to Baltic Proper populations during the microcosm experiments. Average relative abundance in percentage of replicate microcosms together with maximum relative abundance in parenthesis is provided. OTUs recruited from being rare *in situ* (i.e., <0.1% in relative abundance) are indicated with asterisk (^∗^). Alpha., Alphaproteobacteria; Actino., Actinobacteria; Bact., Bacteroidetes; Beta., Betaproteobacteria; Cyano, Cyanobacteria; Gamma., Gammaproteobacteria; Verr., Verrucomicrobia.

§= Only a single replicate, see Table S1.

## Results

### Initial Environmental Conditions and Nutrient Limitation Bioassay

When sampling the seawater for culture media,* in situ* temperature was 15.5 and 15.8°C and salinity was 7.2 and 3.6 for station BAL and BOT, respectively. When sampling the inoculum for initiating the transplant experiment, temperature was 14.8 and 16.7°C and salinity was 7.2 and 3.6, for station BOT and BAL, respectively. Nitrate and ammonium concentrations were about 1.5 times higher and phosphate around 2 times lower at BOT (0.19, 0.83, and 0.06 μM, respectively) compared to BAL (0.12, 0.56, and 0.11 μM, respectively). TOC concentrations were initially different between stations with 3.96 and 4.39 mgL^-1^ for BAL and BOT, respectively. Although nutrient levels were different between BAL and BOT, bacterial nutrient limitation bioassays showed that bacterial growth was not limited by organic carbon or inorganic nutrients at any of the two stations within the time frame of the 48 h experiment (Figure S2).

### Transplant Experiment

**FIGURE 3:**
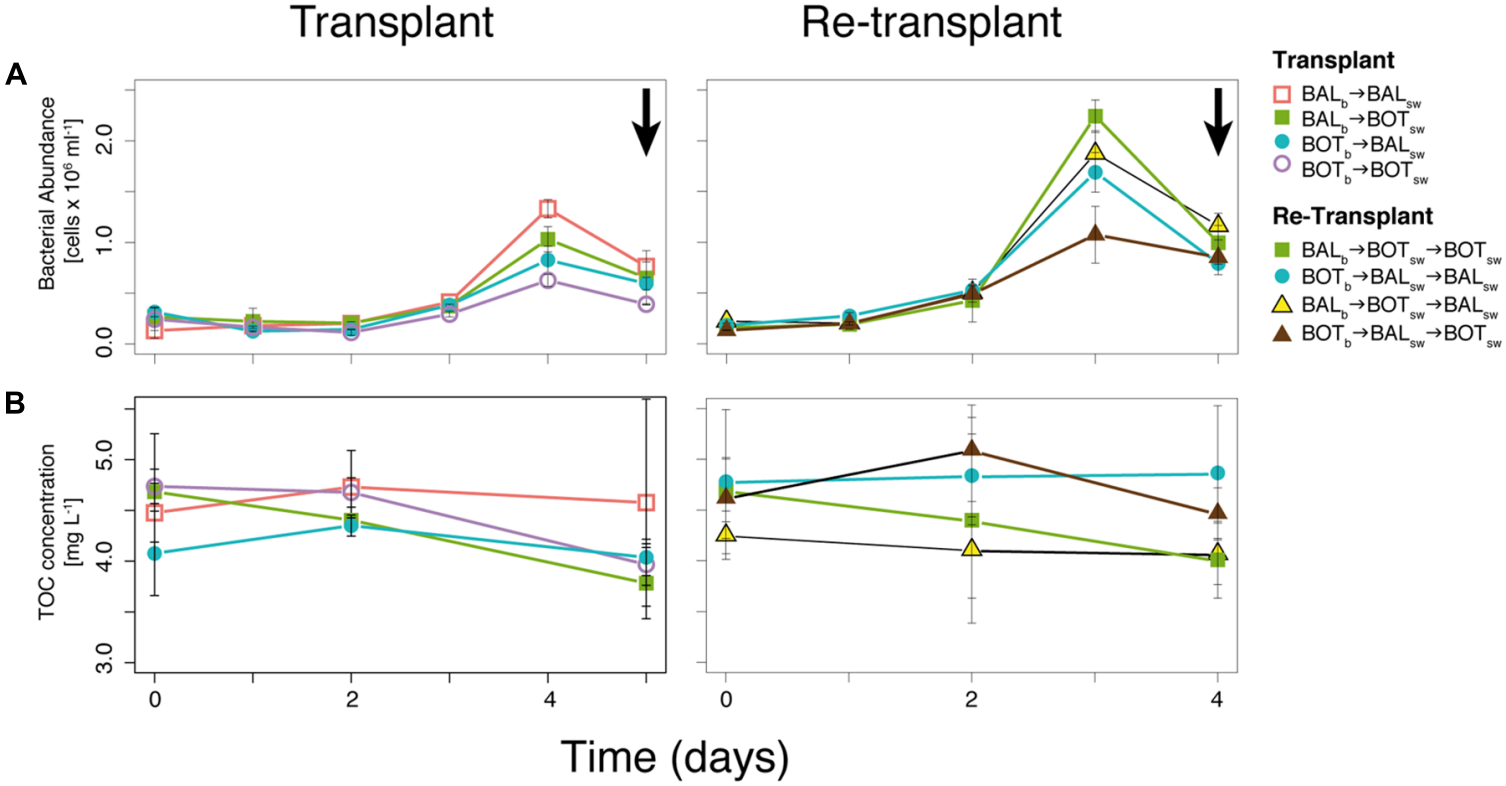
**Bacterial abundance **(A)** and total organic carbon (TOC) concentrations **(B)** during transplant and re-transplant experiments.** Error bars denote SDs for replicate microcosms. Arrows in **(A)** indicate when samples for bacterioplankton community composition were collected.

In the transplant experiment, bacterial abundance increased in all microcosms until day 4 (**Figure 3A**). The BOT_b_ → BOT_sw_ treatment resulted in lower abundance (0.6 × 10^6^ cells ml^-1^) on day 4 compared to BAL_b_ → BAL_sw_ (1.3 × 10^6^ cells ml^-1^; **Figure 3A**). Bacteria in BAL_b_ → BOT_sw_ reached slightly higher abundance than in BOT_b_ → BOT_sw_, (0.8 × 10^6^ cells ml^-1^) on day 4 (**Figure 3A**). TOC concentrations decreased in all microcosms from day 2 to 5 (**Figure 3B**). BAL_b_ → BOT_sw_ microcosms showed a steady decrease in TOC concentrations from 4.75 mg L^-1^ at the start of the experiment to 3.9 mg L^-1^ on day 5 (**Figure 3A**).

**FIGURE 4:**
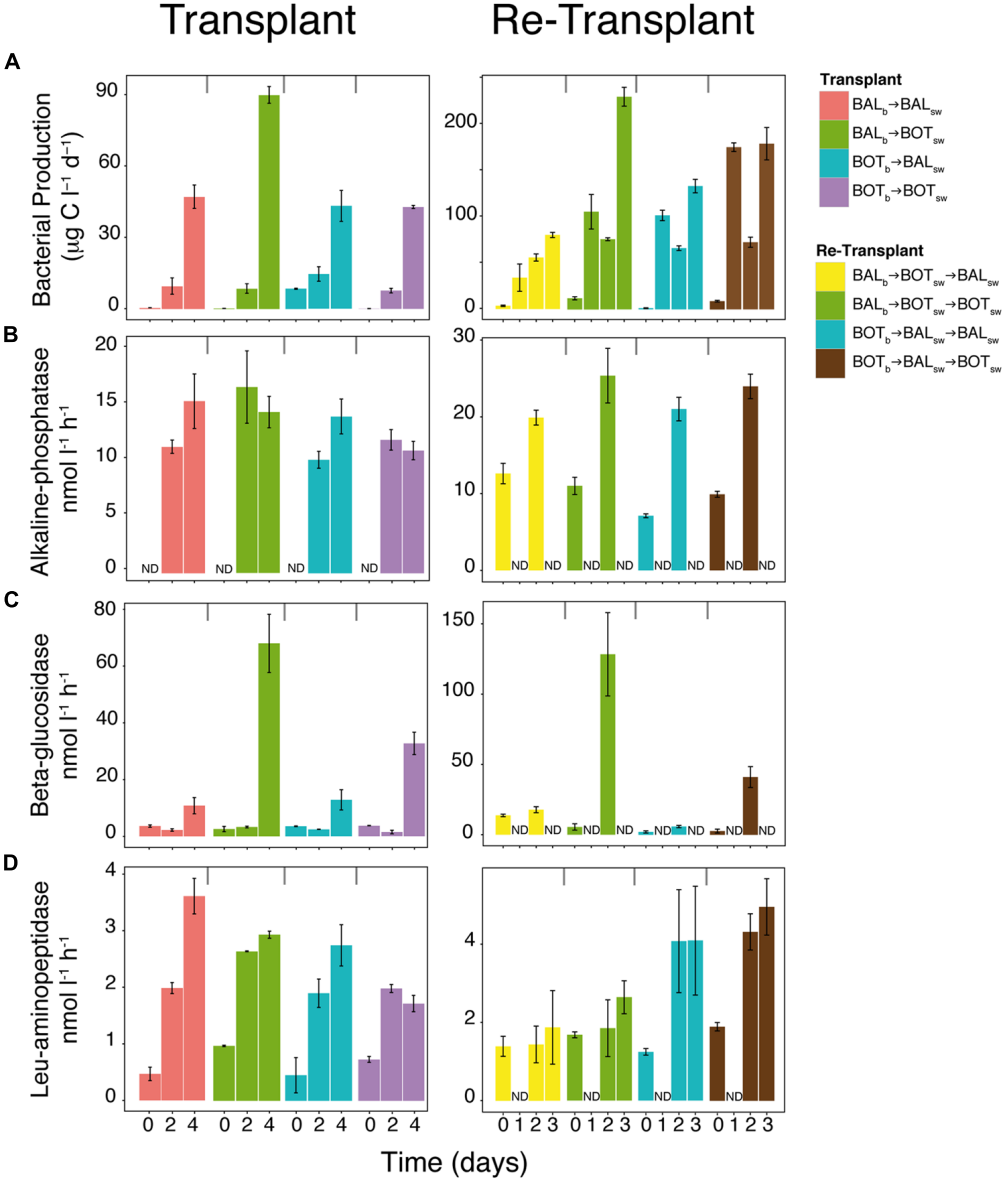
**Measures of bacterial activity in the transplant and re-transplant experiments.** Bacterial heterotrophic production **(A)** and extracellular enzymatic activities **(B–D)**. Error bars denote SDs for quadruplicate technical replicates from biological triplicate microcosms. ND, not determined.

Bacterial production increased in all microcosms during the experiment and reached nearly twice as high levels in BAL_b_ → BOT_sw_ on day 4 (90 μg C L^-1^ d^-1^; **Figure 4A**) compared to the other microcosms (Tukey’s test, *p* = 0.001, *n* = 11). Alkaline-phosphatase activity reached similar levels (10–15 nmol L^-1^ h^-1^) in all microcosms (**Figure 4B**). In contrast, beta-glucosidase activity remained low in the beginning of the experiment but on day 4 increases were observed, with three to sixfold higher responses for both BAL_b_ → BOT_sw_ and BOT_b_ → BOT_sw_ compared to the other microcosms (**Figure 4C**; Tukey’s test, *p* = 0.01, *n* = 11). Leucine-aminopeptidase activity generally increased nearly fourfold during the experiment although levels were variable between treatments (**Figure 4D**).

**FIGURE 5:**
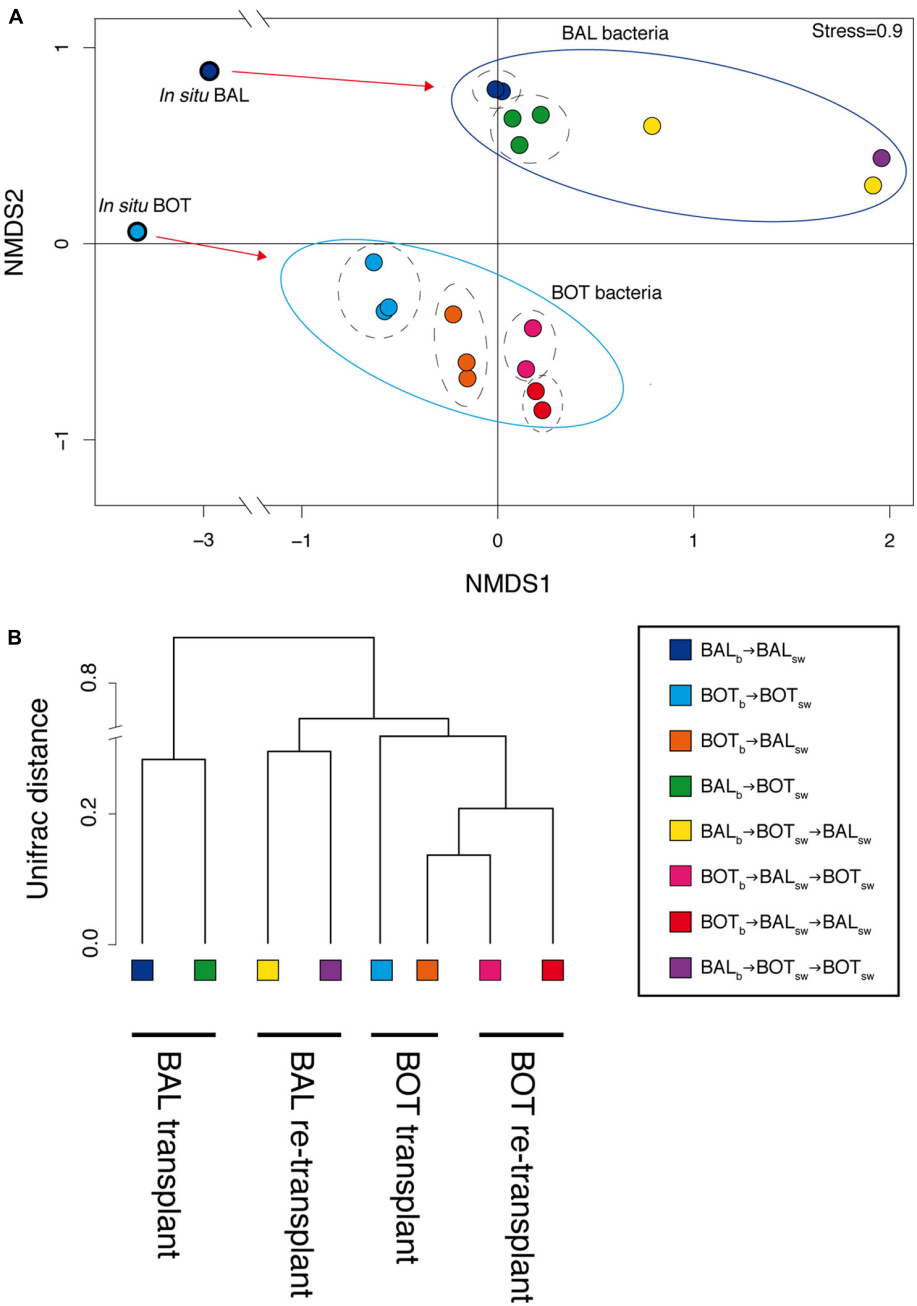
**Comparison of beta-diversity between replicate microcosms with nMDS ordinance calculated from Bray–Curtis distance estimation **(A)** and a dendrogram visualizing Unifrac distances **(B)** using 97% 16S rRNA sequence similarity**.

Analysis of bacterial community composition by nMDS showed a visual clustering of samples largely determined by the source of the inoculum that is either Baltic Proper or Bothnian Sea bacteria (**Figure 5A**). Further, there was a pronounced differentiation between the *in situ* samples and the bacterial communities that developed in the microcosms, but also between communities growing in water from different geographical origin. Thus, BAL_b_ → BAL_sw_ or BAL_b_ → BOT_sw_ microcosm samples clustered separately from each other, and BOT_b_ → BOT_sw_ or BOT_b_ → BAL_sw_ clustered separately (**Figure 5A**). Unifrac analysis confirmed these general patterns, separating samples by the inoculum source and by origin of water used for growth medium (**Figure 5B**). The separation between bacterial inocula, i.e., BAL_b_ vs. BOT_b_, was statistically significant (PERMANOVA, *p* = 0.001, *n* = 18). Moreover, the *in situ* composition was significantly different from that in the microcosms (PERMANOVA, *p* = 0.001, *n* = 20), but there were no significant differences between microcosms in either the transplant and re-transplant experiments.

**FIGURE 6:**
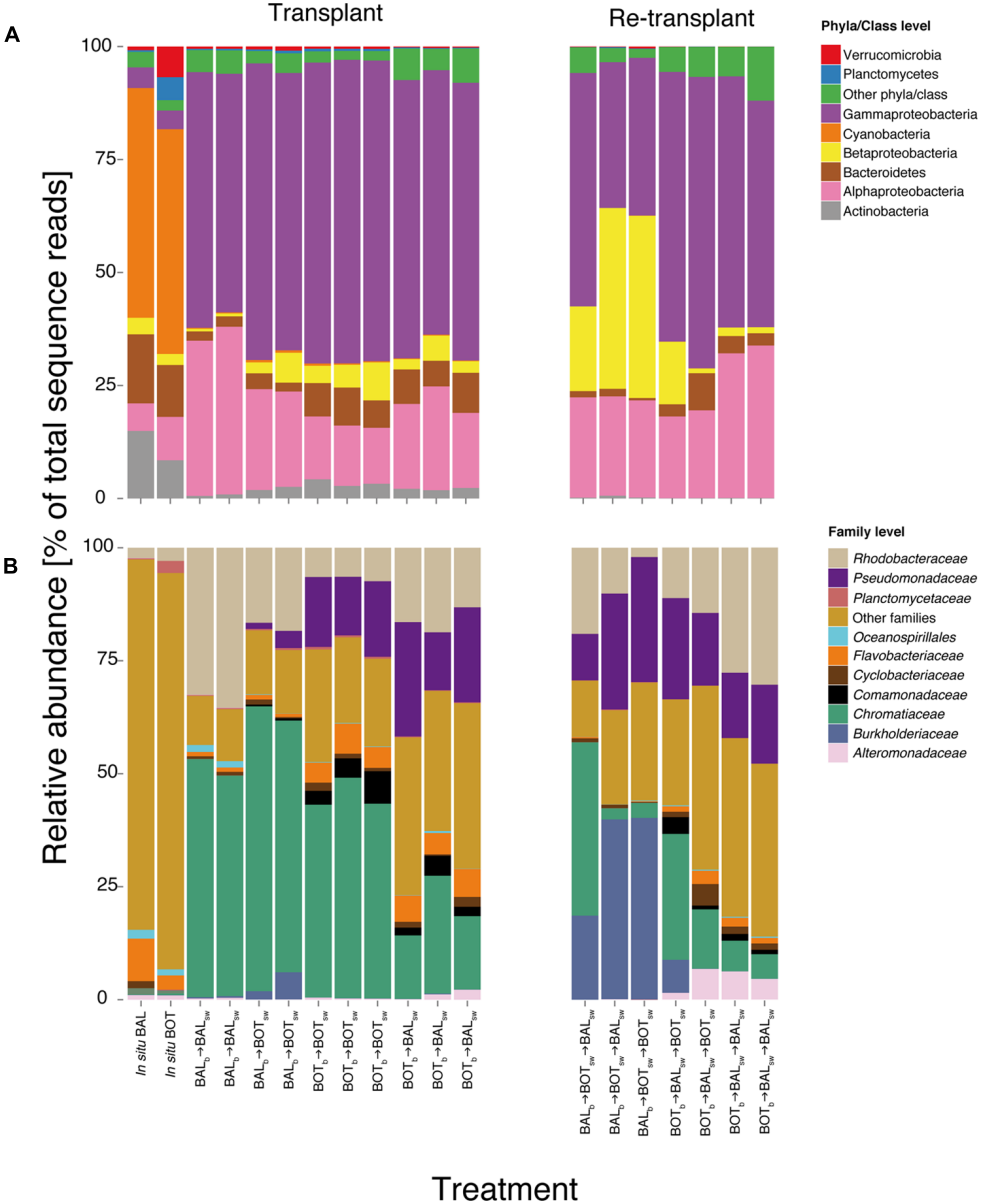
**Bacterial community composition in microcosms at the phyla/class level **(A)** and family level (B).** Relative abundance is calculated from normalized reads, i.e., percent of total sequence reads.

Nevertheless, there were marked changes in community composition between microcosms as seen from pronounced differences both in the presence/absence and in the relative abundance of a variety of bacterial taxa (**Figure 6**). At the phyla/class level, Gammaproteobacteria increased substantially in the experiment compared with their relative abundance in the *in situ* samples, to comprise nearly three quarters of the relative abundance in all microcosms (**Figure 6A**). Cyanobacteria almost disappeared in the microcosms compared to the *in situ* samples, likely resulting from the incubation of microcosms in the dark; accordingly, the diversity within this taxon was higher *in situ*. Among the Gammaproteobacteria, *Chromatiaceae* increased in all microcosms but on average displayed lower relative abundance in BOT_b_ → BAL_sw_ (**Figure 6B**). *Pseudomonadaceae* responded in most microcosms but not in BAL_b_ → BAL_sw_. Alphaproteobacteria had on average higher relative abundance in BAL_b_ → BAL_sw_ compared to the other microcosms. For example, *Rhodobacteraceae* were more abundant in BAL_b_ → BAL_sw_ and BAL_b_ → BOT_sw_ microcosms but also in BOT_b_ → BAL_sw_ compared to BOT_b_ → BOT_sw_. In contrast, Betaproteobacteria reached higher abundance in BAL_b_ → BOT_sw_ and BOT_b_ → BOT_sw_ than in Baltic Proper water, irrespective of the origin of the bacteria (**Figure 6A**). *Comamonadaceae* increased in all microcosms but were nearly absent in BAL_b_ → BAL_sw_ and BAL_b_ → BOT_sw_ (**Figure 6B**). *Flavobacteriaceae* were predominant in BOT_b_ → BOT_sw_ and BOT_b_ → BAL_sw_ microcosms but displayed overall low relative abundance in BAL_b_ → BAL_sw_ and BAL_b_ → BOT_sw_ (**Figure 6B**).

**FIGURE 7:**
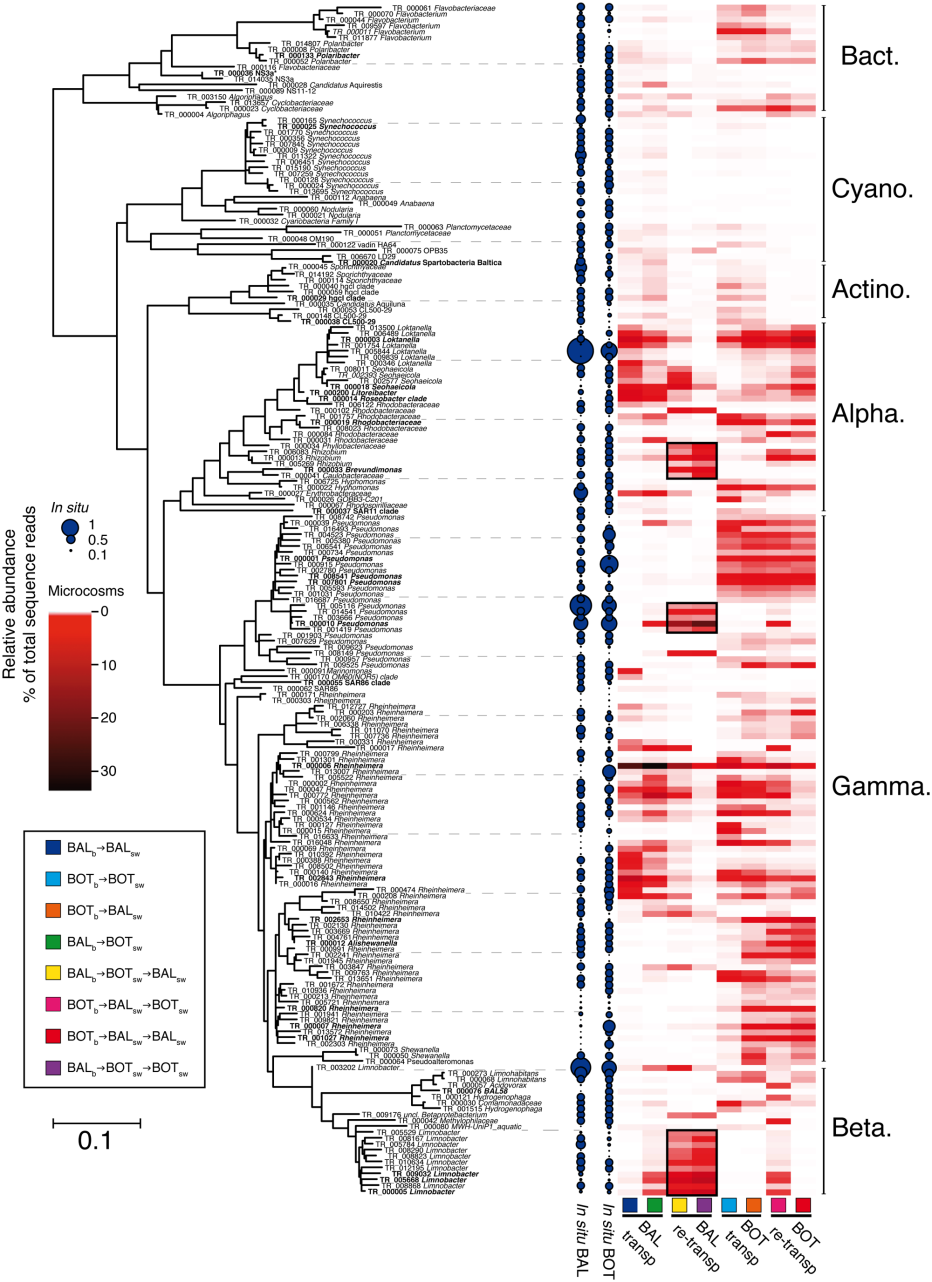
**Maximum-Likelihood tree of 16S rRNA gene sequences obtained from illumina-sequencing.** Heatmaps indicate average relative abundance (percent of total sequence abundance) for the 200 most abundant OTUs in microcosm replicates. OTUs marked in bold are detailed in ****Table 2**** and in the text. Black squares in heatmap denote priming-effect, i.e., OTUs that were triggered in the transplant experiment with continued increases in relative abundance during the re-transplant experiment.

**Figure 7** shows a summary of the response of the 200 most abundant individual populations (i.e., OTUs defined by 97% 16S rRNA gene identity), together representing 82% of total sequence reads. Further detail on particularly important OTUs is given in **Table 2**. Members of bacterial clades that typically are abundant in the Baltic Sea, such as SAR11 (TR_00037), SAR86 (TR_00055), *Synechococcus* (TR_00025), hgcI (TR_00029), and NS3a (TR_00036) were abundant (>1% relative abundance) or common (0.1–1% relative abundance) in our *in situ* samples and did not increase in relative abundance in any microcosms (**Figure 7**; **Table 2**). Nevertheless, among the OTUs that increased in relative abundance in the experiments, a majority (158 OTUs) was found to be common and a few (5 OTUs) were even found to be abundant *in situ* (see OTUs indicated by larger blue filled circles in **Figure 7**). For example, among the alphaproteobacterial OTUs, an OTU affiliated with the *Roseobacter* clade that was abundant in the Baltic Proper *in situ* sample, responded in the transplant experiment. This *Roseobacter* OTU TR_000014 was abundant in BAL_b_→BAL_sw_ microcosms at a relative abundance around 5.2% but reached an elevated relative abundance (2.7%) also in BAL_b_ → BOT_sw_ (**Figure 7**; **Table 2**). We also note that an unclassified *Rhodobacteraceae* OTU (TR_00019) was abundant in BAL_b_ → BAL_sw_, but that this OTU was low in BOT_b_ → BOT_sw_.

Regarding Gammaproteobacteria, three *Pseudomonas* OTUs (TR_08541, TR_07801, TR_00001) were absent in BAL_b_ → BAL_sw_ microcosms and rare *in situ* but became abundant in microcosms with Bothnian Sea bacteria (**Figure 7**; **Table 2**). Sixty populations affiliated with the *Rheinheimera* genus were found among the 200 most abundant OTUs and displayed highly variable patterns of occurrence in the experiments (**Figure 7**). These *Rheinheimera* populations were particularly abundant in transplanted communities and responded both in BAL_b_ → BOT_sw_ and BOT_b_ → BAL_sw_ microcosms (**Figure 7**; **Table 2**). Thus, for example, *Rheinheimera* OTU TR_00006 was highly abundant in BAL_b_ → BAL_sw_ at 26.8% relative abundance and increased to 36.2% in BAL_b_ → BOT_sw_. At the other side of the spectrum, four *Rheinheimera* OTUs (TR_02653, TR_00007, TR_01027, TR_00820) were absent in BAL_b_ → BAL_sw_. These *Rheinheimera* OTUs had low abundance in BOT_b_ → BOT_sw_ but increased to a few percent in BOT_b_ → BAL_sw_ (**Figure 7**; **Table 2**).

### Re-Transplant

Bacterial abundance was higher in most re-transplant microcosms compared to transplant microcosms. However, bacteria in BOT_b_ → BAL_sw_ → BOT_sw_ microcosms reached much lower abundance on day 3 (1.0 × 10^6^ cells ml^-1^) compared to the highest peak (2.2 × 10^6^ cells ml^-1^) for BAL_b_ → BOT_sw_ → BOT_sw_ (**Figure 3B**). TOC concentrations decreased in most re-transplant microcosms over time, except in BOT_b_ → BAL_sw_ → BAL_sw_.

In the re-transplant experiment, bacterial production increased strongly in all microcosms and was generally about twice as high compared to the transplant experiment (**Figure 4A**). With some variability over time, the highest levels were reached in BAL_b_ → BOT_sw_ → BOT_sw_ and BOT_b_ → BAL_sw_ → BOT_sw_ (230 μg C L^-1^ d^-1^ and 180 μg C L^-1^ d^-1^, respectively) compared with the other microcosms (**Figure 4A**; Tukey’s test, *p* = 0.001, *n* = 10). Alkaline phosphatase increased over time from around 10 to 22 nmol L^-1^ h^-1^ over 2 days in all microcosms. As in the transplant experiment, beta-glucosidase activity increased more than 20-fold for both BAL_b_ → BOT_sw_ → BOT_sw_ and BOT_b_ → BAL_sw_ → BOT_sw_ microcosms, while only small changes were observed in BOT_b_ → BAL_sw_ → BAL_sw_ and BAL_b_ → BOT_sw_ → BAL_sw_ (Tukey’s test, *p* = 0.001, *n* = 10). For leucine-aminopeptidase, BOT_b_ → BAL_sw_ → BOT_sw_ and BOT_b_ → BAL_sw_ → BAL_sw_ had twice as high activity, around 4 nmol L^-1^ h^-1^, compared to BAL_b_ → BOT_sw_ → BAL_sw_ on day 2 and 3 (**Figure 4A**).

Bacterial community composition analysis showed that re-transplants pushed the system further compared to the transplant experiment, while at the same time the visual clustering of samples became more variable (**Figure 5A**). When incorporating phylogenetic placement and average relative abundances between replicate microcosms, Unifrac analysis resolved the differentiation between microcosms by decreasing some of the variation observed in the nMDS analysis (**Figure 5B**).

In the re-transplant experiment, Betaproteobacteria reached overall higher relative abundance in BAL_b_ → BOT_sw_ → BAL_sw_ and BAL_b_ → BOT_sw_ → BOT_sw_ (**Figure 6A**). Concomitantly, Alphaproteobacteria were more important in BOT_b_ → BAL_sw_ → BAL_sw_ compared to the other microcosms. *Alteromonadaceae* became abundant in BOT_b_ → BAL_sw_ → BAL_sw_ and BOT_b_ → BAL_sw_ → BOT_sw_, continuing an increase triggered already in BOT_b_ → BAL_sw_ (**Figure 6B**). Similarly, *Burkholderiaceae* continued to increase in relative abundance in BAL_b_ → BOT_sw_ → BAL_sw_ and BAL_b_ → BOT_sw_ → BOT_sw_, after being triggered upon growth in BAL_b_ → BOT_sw_ (**Figure 6B**). From here on we refer to this triggering of populations from transplant to re-transplant as a “priming effect.”

Several of the OTUs that increased in the re-transplant experiment were not only rare *in situ* but also remained undetected or rare during the transplant experiment (**Figure 7**). For example, *Brevundimonas* OTU TR_000033 accounted for around 5% of the assemblage in the BAL_b_ → BOT_sw_ → BAL_sw_ and BAL_b_ → BOT_sw_ → BOT_sw_ microcosms (**Figure 7**; **Table 2**) but was below the detection limit in the other microcosms and during the transplant experiment. A priming effect was observed for three *Limnobacter* OTUs (TR_005668, TR_000005, TR_009032) that were rare *in situ* and virtually absent during the transplant experiment, except in BAL_b_ → BOT_sw_. These OTUs increased substantially in BAL_b_→BOT_sw_→BAL_sw_ and BAL_b_ → BOT_sw_ → BOT_sw_. The *Pseudomonas* OTU TR_000010 was also primed already in BAL_b_ → BOT_sw_ microcosms and further increased over 10-fold in the re-transplant BAL_b_ → BOT_sw_ → BAL_sw_ and BAL_b_ → BOT_sw_ → BOT_sw_ microcosms. Similarly, priming effects were observed for three *Rhizobium* OTUs (TR_006083, TR_000013, and TR_005269) in BAL_b_ → BOT_sw_ → BAL_sw_ and BAL_b_ → BOT_sw_ → BOT_sw_. Further, a *Loktanella* population (TR_000003) that responded in all microcosms during the transplant experiment was only found in BOT_b_ → BAL_sw_ → BAL_sw_ during the re-transplant experiment (**Table 2**). In the re-transplant experiment, much lower levels were observed of the *Roseobacter* OTU TR_000014 (0.6% in BAL_b_ → BOT_sw_ → BAL_sw_) compared to the initial transplant (2.7%; **Figure 7**; **Table 2**).

### Diversity

Lower levels of Shannon and Chao1 indexes were detected in BAL_b_ → BOT_sw_ compared to BAL_b_ → BAL_sw_ microcosms (**Table 3**). On the other hand, Shannon diversity reached the highest value in BOT_b_ → BAL_sw_ compared to all other microcosms and the *in situ* samples. Alpha diversity levels remained relatively low in the BAL_b_ → BOT_sw_ → BAL_sw_ microcosms and decreased further in BAL_b_ → BOT_sw_ → BOT_sw_.

**Table 3:** Shannon and Chao1 indexes ± SD “–” indicate lack of replicates.

Treatment	Shannon	Chao1
*In situ* BAL	4.59 –	1247.03 –
*In situ* BOT	4.97 –	1296.72 –
BAL_ b_→BAL_sw_	4.60 ± 0.11	2352.21 ± 164.45
BOT_ b_→BOT_sw_	4.68 ± 0.02	2221.73 ± 271.36
BOT_ b_→BAL_sw_	5.06 ± 0.10	2195.57 ± 317.30
BAL_ b_→BOT_sw_	3.55 ± 0.15	1383.66 ± 83.91
BAL_ b_→BOT_sw_→BAL_sw_	3.62 ± 0.05	1248.68 ± 106.98
BOT_ b_→BAL_sw_→BOT_sw_	4.65 ± 0.09	1843.28 ± 95.054
BOT_ b_→BAL_sw_→BAL_sw_	4.68 ± 0.12	2045.08 ± 759.73
BAL_ b_→BOT_sw_→BOT_sw_	3.41 –	1222.18 –

### Linking Bacterial Community Composition and Phylogeny with Bacterial Community Functioning

**FIGURE 8:**
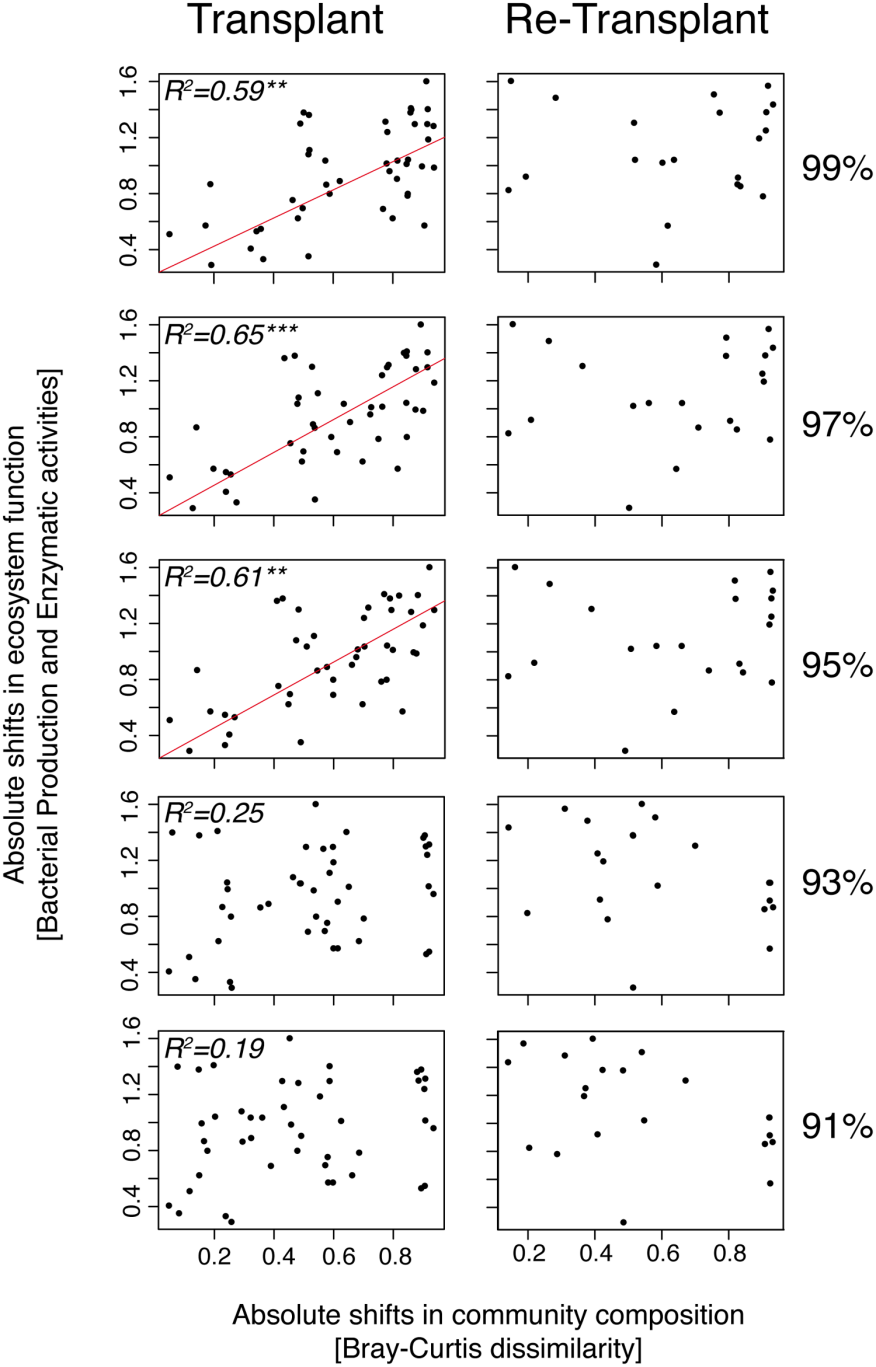
**MANTEL tests and plots of distance matrices of absolute shifts in community composition measured by Bray–Curtis distance estimation versus absolute shifts in bacterial community functioning (pooled bacterial production and enzyme activities) measured by Canberra distance estimation.** Bray–Curtis dissimilarity matrices of community composition were performed at different cluster levels (99, 97, 95, 93, and 91%) of 16S rRNA gene identity. All microcosms are compared with each other. Red lines indicate significant correlations and *R*^2^ values above 0.5. Asterisks denote the significance levels; ^∗∗^*p* < 0.001, ^∗∗∗^*p* < 0.0001.

To determine if specific bacterial taxa could be associated with responses in enzymatic activities we performed PERMANOVA tests (Table S2). Although we found significant correlations between enzyme activities and, e.g., *Alteromonadaceae* (PERMANOVA, *p* = 0.01, *R*^2^= 0.28, *n* = 18), and *Chromatiaceae* (PERMANOVA, *p* = 0.01, *R*^2^= 0.26, *n* = 18), such correlations explained typically less than 20% of the variance (Table S2). Interestingly though, several taxa were significantly correlated with either beta-glucosidase or leucine-aminopeptidase but not with alkaline-phosphatase. Next, we analyzed bacterial community functioning (i.e., collective differences in bacterial production and enzyme activities) versus community composition clustered at different phylogenetic levels. In the transplant experiment, absolute shifts in community composition were significantly correlated with absolute shifts in bacterial community functioning, especially at the 97% 16S rRNA gene sequence cluster identity level (MANTEL, *p* = 0.001, Pearson *R*^2^= 0.65, *n* = 17; **Figure 8**). The correlation between bacterial community composition and bacterial community functioning was also strong at the 99 and 95% cluster level (MANTEL, *p* < 0.01, Pearson *R*^2^ = 0.59–61, *n* = 17) but became weak and insignificant at lower taxonomic resolution (<95%). The absolute shifts in community composition and absolute shifts in bacterial community functioning in the re-transplant experiment were not significantly correlated (**Figure 8**).

## Discussion

### Bacterial Responses in Community Functioning

In our microcosm experiments we used transplants of bacterial assemblages to investigate connections between bacterioplankton community composition and metabolic plasticity in response to contrasting environmental conditions between the different Baltic Sea basins. Our experimental manipulations showed substantial differences between microcosms in terms of bacterial abundance (**Figure 3A**), organic carbon utilization (**Figure 3B**), bacterial heterotrophic production (**Figure 4A**), and beta-glucosidase activity (**Figure 4C**), indicating that bacterial community functioning changed when bacteria were exposed to water from different geographical origins. For example, when Baltic Proper bacteria were challenged by new environmental conditions found in Bothnian Sea water, we observed increased bacterial production and beta-glucosidase activity. Similar changes in bacterial production and enzyme activity occurred also in re-transplant experiments, reinforcing the role of Bothnian Sea water on the observed metabolic changes.

Our findings indicated that Baltic Proper bacteria show metabolic plasticity when transferred to Bothnian Sea water, as deduced from the observation that activity in these treatments actually increased. This effect was further promoted when transplanted Baltic Proper bacteria were allowed to resume growth in Bothnian Sea water during the re-transplant experiment. The elevated levels of activity for Baltic Proper bacterial communities in changed environmental conditions compared to controls may suggest that higher bacterial activities could be expected in the Baltic Proper in response to climate change induced reductions in salinity and increased terrestrial DOM runoff. However, it is important to consider that results from this study are based on short-term responses to disturbances, whereas the effects of climate change implicate long-term changes in the water-chemistry of the Baltic Sea. Still, disturbance events that in part contribute to the long-term changes, for example massive river runoff events following heavy rainfall or storm induced upwelling of nutrient rich waters, are likely to be more frequent with anthropogenically induced changes in environmental conditions. These findings substantiate and support earlier model data and experimental results from the Baltic Sea, implicating changes from autotrophy toward microbial heterotrophy with increases in riverine outflow due to climate change (Sandberg et al., 2004; Wikner and Andersson, 2012; Degerman et al., 2013; Lefébure et al., 2013).

### Bacterioplankton Community Change

Concurrently with the changes in bacterial community functioning, transplants, and re-transplants of bacterial assemblages between water from different geographical origins also caused changes in the composition of bacterial communities. For example, shifts in composition were accompanied by increased bacterial production and beta-glucosidase activity in transplants with Baltic Proper bacteria growing in Bothnian Sea water. However, the shifts in environmental conditions did not completely transform the communities so that they all became the same, i.e., Baltic Proper communities did not converge to the same structure as Bothnian Sea communities and neither vice versa (**Figure 5**). In accordance with our results, substantial shifts in bacterial community composition also occurred when transplanting bacteria between Baltic Sea and Skagerrak Sea conditions, yet the communities did not become similar (Sjöstedt et al., 2012). These experimental approaches are short-term while *in situ* responses may look very different in the long run, emphasizing the need to carry out longer experiments and *in situ* time-series to elucidate the resistance, resilience, and sensitivity of bacterial communities responding to environmental disturbances. Nevertheless, a multitude of experimental and *in situ* approaches in coastal waters have established that bacterial community structure is sensitive to environmental disturbances, e.g., changes in terrestrial DOM (Kisand and Wikner, 2003; Rochelle-Newall et al., 2004; Kisand et al., 2008; Teira et al., 2009; Grubisic et al., 2012; Rocker et al., 2012) and salinity (Langenheder et al., 2003; Kaartokallio et al., 2005; Sjöstedt et al., 2012). Taken together, our data indicate distinct responses and links between bacterial community composition and community functioning resulting from exposure to seawater from the northern vs. southern Baltic Sea basins.

We have no immediate knowledge as to the specific chemical characteristics in the seawater from the Baltic Proper and Bothnian Sea that could have driven changes in bacterioplankton community structure in our experiments. Salinity is a critical factor in regulating bacterial community composition (Langenheder et al., 2003; Lozupone and Knight, 2007; Herlemann et al., 2011; Sjöstedt et al., 2012). A recent metagenomic analysis indicated genomic features that may contribute to such regulation (Dupont et al., 2014). Some of these features were indicative within the same narrow range of salinities (salinity 3.6–7.2) that characterize our studied waters. Moreover, salinity can regulate bacterial community functioning, and low salinity may have a negative influence on the growth and activity of marine bacterioplankton degrading terrigenous carbon (Langenheder et al., 2003; Kisand et al., 2008). Although bacteria can degrade allochthonous DOM (Rochelle-Newall et al., 2004; Rocker et al., 2012), autochthonously produced DOM is often more efficiently utilized due to its less refractory nature (Kritzberg et al., 2004). However, allochthonous DOM can lead to higher respiration and not be incorporated into biomass (Fasching et al., 2014). The discharge of allochthonous DOM is higher into the northern basins of the Baltic compared to the Baltic Proper (Omstedt et al., 2014). Furthermore, inorganic nutrient concentrations could have influenced the bacterial dynamics in our experiments. However, nutrient limitation bioassays with *in situ* samples indicated that the investigated communities were not directly limited by nutrient availability (Figure S2). Further, physicochemical factors, such as limitation of trace metals (Church et al., 2000), or top–down effects, such as protist grazing, or virus predation (Jürgens et al., 1999; Bouvy et al., 2011), may contribute to promoting changes in community structure and bacterial community functioning of transplanted bacterial communities. In addition, it is also important to consider that seasonal and inter-annual variation in environmental conditions, from, e.g., phytoplankton blooms, result in a succession of bacterioplankton populations and a wide spectrum of responses in abundances (Andersson et al., 2010; Lindh et al., 2015). Anthropogenically induced changes (in e.g., temperature) may influence such seasonal patterns, which could complicate interpretations of responses to precipitation patterns of bacterioplankton populations.

In our study, particular bacterial groups and populations showed distinct responses to water from different geographical origin in the experiments (**Figure 6**; **Table 2**). Thus, although there were pronounced changes in all microcosms, specific treatment effects resulted in communities that were distinct from one another at the end of the experiment. Community composition change due to environmental disturbances often results in the recruitment of rare OTUs that become abundant, as demonstrated both experimentally and *in situ* (Campbell et al., 2011; Sjöstedt et al., 2012; Alonso-Saez et al., 2014). However, it is noteworthy that among the 200 most abundant OTUs that responded in the microcosms at the end of the experiments, a few OTUs were actually abundant (>1% relative abundance, *n* = 5), while the grand majority were common (0.1–1% relative abundance, *n* = 158) *in situ*. In contrast, only 33 OTUs that responded in the experiments were initially rare (<0.1% relative abundance). These findings show that not all responsive OTUs represented initially rare copiotrophic populations stimulated by artificial “bottle-effects” but that common populations *in situ* are particularly responsive to environmental disturbances.

Among the initially rare populations several *Limnobacter* OTUs increased in abundance when Baltic bacteria were transferred to Bothnian Sea water, and several *Pseudomonas* OTUs found among the Bothnian Sea bacteria proliferated in Baltic Proper water; this indicated replacement of populations. Also adjustment of bacterial populations to the experimental disturbances was observed among the bacterial populations (**Figure 7**; **Table 2**). In particular, one *Roseobacter* OTU was not only abundant *in situ* and in control microcosms, but also in transplants of Baltic proper bacteria to Bothnian Sea water. In addition, *Rheinheimera* populations were highly variable between microcosms, indicating population adjustment (**Figure 7**; **Table 2**). Collectively, our transplant and re-transplant experiments suggest a balance of adjustment and replacement effects when bacteria encounter distinct water conditions from different geographical origin.

### Priming Effect

Recruitment of rare bacteria as a response to changes in environmental conditions can result from proliferation of both specialist and generalist populations (Mills and Mallory, 1987; Atlas et al., 1991; Campbell et al., 2011; Lennon and Jones, 2011). Some bacterial taxa triggered in the transplant experiment, e.g., *Alteromonadaceae* and *Burkholderiaceae* OTUs, continued to increase in relative abundance during the re-transplant experiment in both types of seawater media, as a result of a “priming effect.” Such priming seems to have resulted from the initial triggering of increases in abundance of a limited number of populations by exposure to water from a different location; and this initial growth stimulation then continued upon transfer also to waters from different basins. This response may result from challenging a bacterial community that is not immediately resilient but rather reward generalist OTUs that were successful in transplants. Therefore, it would be highly interesting to study the resilience potential of disturbed bacterial communities over longer time scales, either in long-term experiments or over several years *in situ* to elucidate the pace and frequency at which specific populations recover their abundances or the bacterial community returns to previous undisturbed structure.

### Diversity

An important ecological mechanism in nature is the insurance hypothesis or portfolio effect that balances negative (i.e., sensitive species) and positive effects (i.e., responsive species) simply by carrying a large number of taxa (Loreau, 2000; Allison and Martiny, 2008). This mechanism can result in a scenario, where bacterial community composition changes while maintained or even increased bacterial community functioning can be observed compared to the undisturbed community. The insurance hypothesis is intriguing; especially in relation to future climate change and the growing awareness of its substantial long term effects on biodiversity in all parts of the food-web in marine environments across the globe (Worm et al., 2006; Awasthi et al., 2014). Although richness effects on bacterial community functioning may be less important under current environmental conditions, they are likely to become important for handling future environmental disturbances (Loreau, 2000; Bell et al., 2005; Awasthi et al., 2014).

In our experiments, bacterial responses to experimental disturbances heavily influenced alpha diversity. Shannon and Chao1 levels were substantially lower in all microcosms with Baltic Proper bacteria except the controls (**Table 3**). Lower alpha diversity due to transplants and re-transplants with Baltic Proper bacteria could suggest that only few populations are able to cope with the changes in environmental conditions to which they were exposed. Alternatively, a few populations that were highly competitive under the new seawater conditions could increase in relative abundance to become dominant. In fact, lower alpha diversity was found when metabolic activity was high and community composition changed substantially, as exemplified by Baltic Proper bacteria growing in Bothnian Sea water. These data suggest that a portfolio effect likely aided the response of bacterial community composition and bacterial community functioning in the transplant experiment (Wittebolle et al., 2009; Awasthi et al., 2014). However, the resulting low alpha diversity due to transplants possibly led to a chaotic response in community composition and a more variable effect on metabolic activity during the re-transplant experiment, suggesting that environmental disturbances such as increased riverine discharge may render disturbed communities highly sensitive. Taken together, many OTUs in the Baltic Proper seem to be well suited for Bothnian sea-like environmental conditions; that is future predicted increases in terrigenous organic matter and lower salinity, but at the cost of overall lower alpha diversity and potentially a reduced responsiveness to added environmental change.

### Bacterial Community Functioning

The current debate of functionally redundant versus non-redundant bacterial communities is complex (Loreau, 2004; Wohl et al., 2004; Allison and Martiny, 2008; Comte and Del Giorgio, 2011; Miki et al., 2014). However, transplant and re-transplant experiments can be used to address some of the fundamental questions regarding the role of community composition for bacterial responses in metabolic activity (**Figure 8**; Table S2). In the transplant experiment, we observed a positive relationship between absolute shifts in community composition and absolute shifts in bacterial community functioning (explaining >60% of the variance, depending on phylogenetic scale). Interestingly this relationship was most prominent at 97% 16S rRNA gene sequence identity and only observed at ≥95%. At lower taxonomical resolution, community composition and bacterial community functioning were not correlated. The lack of correlation at lower taxonomic resolution thus resulted from the counterbalancing of differential responses among individual populations within the same major taxon and highlights the importance of analyzing specific responses to environmental disturbances at a detailed phylogenetic level.

In the re-transplant experiment this relationship was lacking regardless of phylogenetic scale, which would lead to the conclusion that bacterial assemblages were functionally redundant. However, it is important to note here that the relationship between community composition and bacterial community functioning breaks down in the experiment with continued experimental forcing (i.e., in the re-transplant experiment) in which the bacterial community had already gone through a pronounced succession from the original time zero. This could indicate that successional progression temporarily offsets perceived relationships between bacterial community composition and functioning. In other words, interpretations of levels of redundancy, and hence the importance of species richness in the context of the insurance hypothesis/portfolio effect (Loreau, 2000; Allison and Martiny, 2008), could be heavily distorted both by the complexity of natural bacterial assemblages and by the inability to adequately determine successional stages of investigated communities. These findings indicate the efficacy of combining longer experiments with high taxonomical resolution (≥97% 16S rRNA gene identity) analyses for interpreting distribution patterns of individual bacterial populations in relation to environmental forcing. Ultimately, such analyses have the potential to identify causal relationships between bacterial community composition and functioning.

### Conclusion

According to our hypothesis, bacterial community composition and functioning would change after both transplantation and re-transplantation disturbances, following the replacement scenario. Indeed, this hypothesis was confirmed in the transplant experiment, such that the changes in community composition accounted for by responsive bacterial populations were reflected also in adjustment of bacterial activities. However, when adding a continued experimental forcing to the already disturbed community in the re-transplant experiment, the linkage between change in community composition and change in community functioning became disrupted. Rejection of our hypothesis in the re-transplant experiment implies that disturbances caused distinct responses of specialist or generalist bacteria in a manner that was dependent on the successional stage at which the disturbance took place. Our findings further indicate the potential of experimental manipulations to aid interpretations of the adaptability and metabolic plasticity of bacterioplankton communities responding to changes in environmental conditions. Notably, exposure of Baltic Proper bacteria to humic rich/low salinity Bothnian Sea water caused higher metabolic activity, while at the same time inducing shifts in bacterial community structure. This supports recent suggestions that climate change could lead to undesirable long-term shifts toward an increasingly net heterotrophic system in the Baltic Proper. Alterations in precipitation patterns across seasons or years or increased frequency of event driven river runoff episodes may at first result in only subtle changes in community composition or bacterial activities. However, such changes in runoff could have essential priming effects on bacterial community structure that subsequently translate into longer-term changes in bacterial community functioning and biogeochemical process rates.

## Conflict of Interest Statement

The Editor Jürg Brendan Logue declares that, despite being affiliated to the same institution as the author Johanna Sjöstedt, the review process was handled objectively and no conflict of interest exists. The authors declare that the research was conducted in the absence of any commercial or financial relationships that could be construed as a potential conflict of interest.
