# Supplementary material for: Transplant experiments uncover Baltic Sea basin-specific responses in bacterioplankton community composition and metabolic activities
Source: Front Microbiol. 2015 Apr 1;6:223. doi: 10.3389/fmicb.2015.00223 (PMC4381636; doi:10.3389/fmicb.2015.00223)
Supplement: Supplementary file 1 [file data_sheet_1.docx]

***Supplementary Material***

**Transplant experiments uncover Baltic Sea basin-specific responses in bacterioplankton community composition and metabolic activities**

**Markus V. Lindh^1^, Daniela Figueroa^2^, Johanna Sjöstedt^1§^, Federico Baltar^1,3^,Daniel Lundin^1^, Agneta Andersson^3^, Catherine Legrand^1^, Jarone Pinhassi^1^***

^1^Centre for Ecology and Evolution in Microbial model Systems - EEMiS, Linnaeus University, SE-39231 Kalmar, Sweden.

^2^Department of Ecology and Environmental Science, Umeå University, Umeå SE-90187, Sweden.

^3^Department of Marine Sciences, University of Otago, PO Box 56, Dunedin NZ-9054, New Zealand

^§^ Present address: Department of Biology/Aquatic Ecology, Lund University, SE-22362 Lund, Sweden and Department of Ecology and Genetics, Uppsala University, SE-75236 Uppsala, Sweden

*** Correspondence:** jarone.pinhassi@lnu.se

1. **Supplementary Figures and Tables**

**Figure S1.** Experimental procedure. Seawater (sw) from stations BAL and BOT was filtered through 0.2 μm supor filters (PALL Life Sciences) and distributed into 2 l polycarbonate bottles (Nalgene) and autoclaved for seawater media. Unfiltered seawater was then added to each bottle; in transplants unfiltered seawater was added to the opposite media and in re-transplants transplanted water was retransferred to the media equivalent to the original bacterial source. Controls were incubations of unfiltered seawater in the same media as the bacterial source for transplants and continued incubation in changed media for re-transplants. All incubations were done in triplicates at 1:20 ratio.

**Figure S2.** Bacterial nutrient limitation bioassays of *in situ* samples from BAL (A), and BOT (B). Error bars denote standard deviations for quadruplicate measurements. K = Control, C = Glucose (C_6_H_12_O_6_), N = Ammonium (NH_4_Cl), P = Phosphate (NaH_2_PO_4_).

**Table S1.** Number of replicates per *in situ* sample and microcosm treatment.

| **Treatment** | **No. of replicates** |
| --- | --- |
| *In situ* BAL | 1 |
| *In situ* BOT | 1 |
| BALb**🡒**BALsw | 2 |
| BOTb**🡒**BOTsw | 3 |
| BOTb**🡒**BALsw | 3 |
| BALb**🡒**BOTsw | 3 |
| BALb**🡒**BOTsw**🡒**BALsw | 2 |
| BOTb**🡒**BALsw**🡒**BOTsw | 2 |
| BOTb**🡒**BALsw**🡒**BALsw | 2 |
| BALb**🡒**BOTsw**🡒**BOTsw | 1 |

**Table S2.** Summary of PERMANOVA tests of correlations between enzyme activities and specific taxon at phyla/class level (bold face) and examples from family level (italics) with their corresponding *p*-value. Number of OTUs (n) is indicated after each taxon, R^2^ values in parenthesis and degrees of freedom = 17. Asterisk (*) indicates significant correlations. Bgase = Beta-glucosidase, Lapase = Leucine-aminopeptidase and Apase = Alkaline-phosphatase.

| Taxon | Bgase | Lapase | Apase |
| --- | --- | --- | --- |
| All (n=3920) | 0.16 (0.08) | 0.01 (0.15)* | 0.22 (0.07) |
| **Actinobacteria** (n=161) | 0.006 (0.17)* | 0.001 (0.19)* | 0.52 (0.05) |
| **Alphaproteobacteria** (n=604) | 0.16 (0.09) | 0.003 (0.18)* | 0.25 (0.07) |
| *Rhodobacteriaceae* (n=284) | 0.10 (0.10) | 0.01 (0.17)* | 0.21 (0.08) |
| **Bacteroidetes** (n=516) | 0.03 (0.12)* | 0.03 (0.12)* | 0.53 (0.04) |
| *Cyclobacteriaceae* (n=129) | 0.30 (0.07) | 0.20 (0.08) | 0.70 (0.02) |
| *Flavobacteriaceae* (n=202) | 0.01 (0.20)* | 0.03 (0.13)* | 0.19 (0.08) |
| **Betaproteobacteria** (n=333) | 0.17 (0.09) | 0.08 (0.12)* | 0.57 (0.04) |
| *Burkholderiaceae* (n=120) | 0.68 (0.03) | 0.05 (0.12)* | 0.48 (0.05) |
| *Comamonadaceae* (n=116) | 0.21 (0.07) | 0.02 (0.12)* | 0.29 (0.07) |
| **Gammapreoteobacteria** (n=1149) | 0.34 (0.06) | 0.05 (0.12)* | 0.26 (0.07) |
| *Alteromonadaceae* (n=93) | 0.70 (0.04) | 0.01 (0.28)* | 0.26 (0.02) |
| *Chromatiaceae* (n=376) | 0.01 (0.26)* | 0.25 (0.07) | 0.71 (0.03) |
| *Pseudomonadaceae* (n=279) | 0.10 (0.11) | 0.13 (0.10) | 0.45 (0.04) |
| **Planctomycetes** (n=64) | 0.62 (0.03) | 0.23 (0.07) | 0.80 (0.03) |
| *Planctomycetaceae* (n=39) | 0.63 (0.04) | 0.12 (0.09) | 0.93 (0.01) |
| **Verrucomicrobia** (n=110) | 0.04 (0.17)* | 0.34 (0.06) | 0.60 (0.03) |
