# Supplementary figures and images for: Transplant experiments uncover Baltic Sea basin-specific responses in bacterioplankton community composition and metabolic activities
Source: Front Microbiol. 2015 Apr 1;6:223. doi: 10.3389/fmicb.2015.00223 (PMC4381636; doi:10.3389/fmicb.2015.00223)

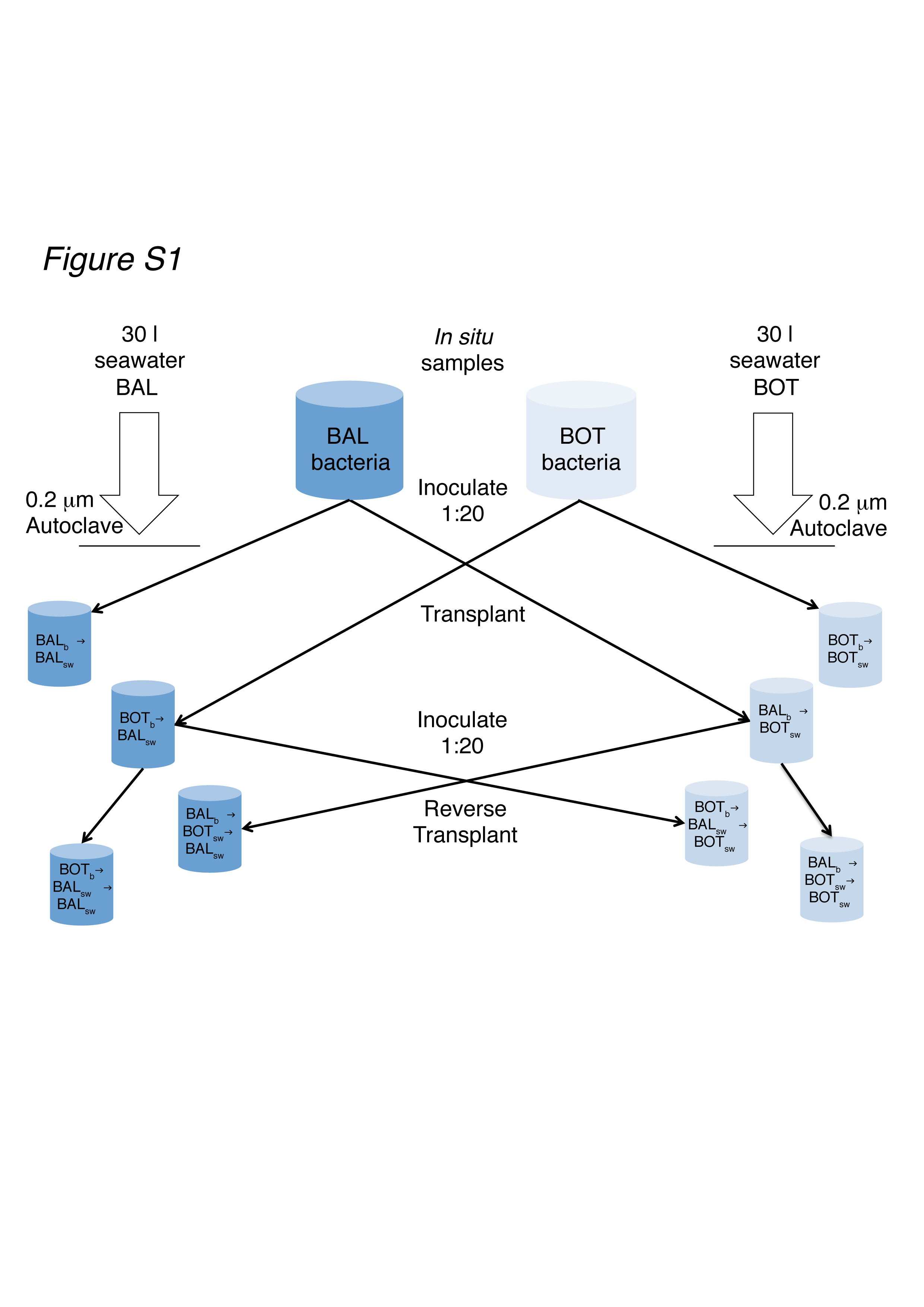

Supplement: Supplementary file 2 [file image_1.tif]

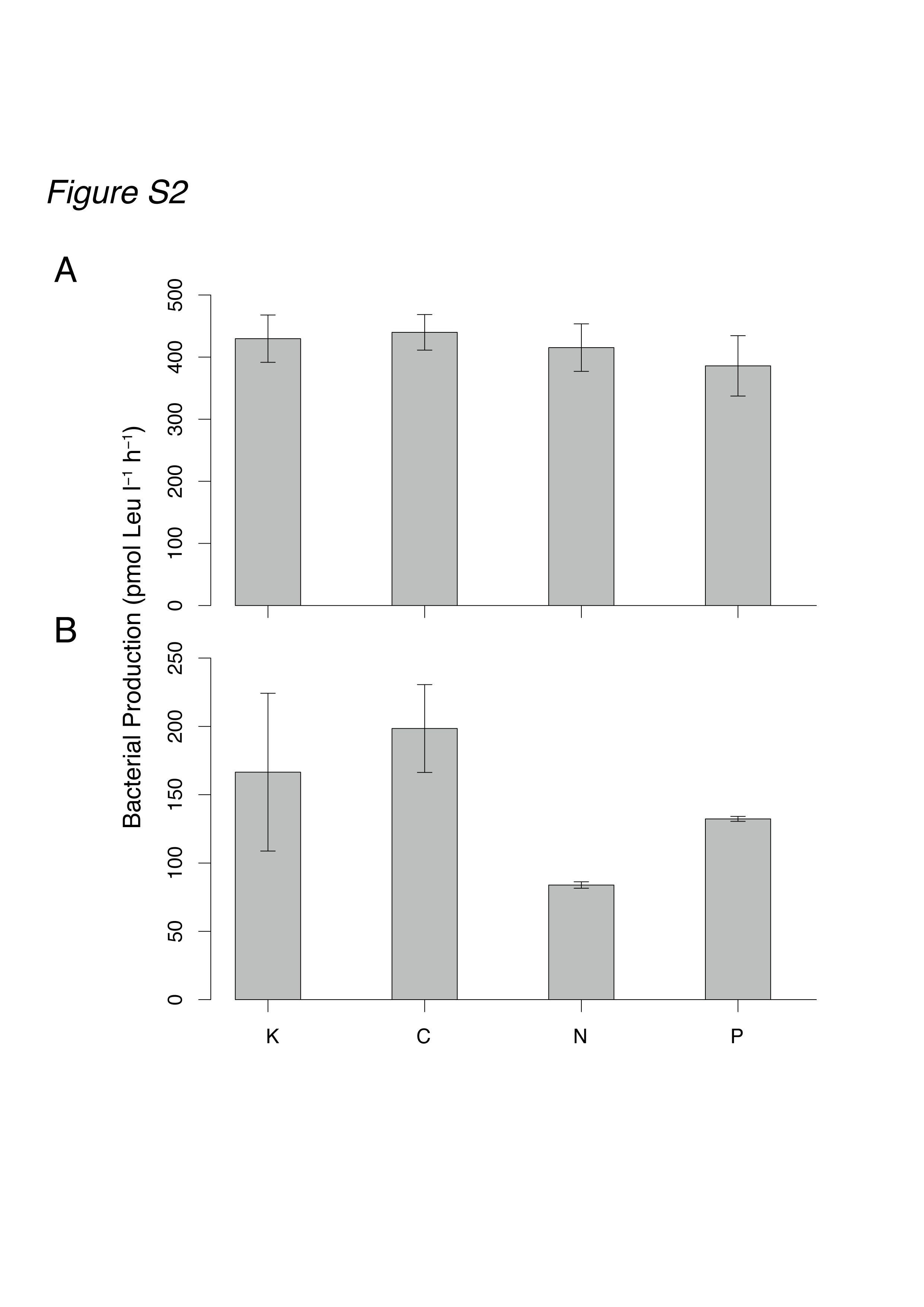

Supplement: Supplementary file 3 [file image_2.tif]
